# Biosafety of biotechnologically important microalgae: intrinsic suicide switch implementation in cyanobacterium *Synechocystis* sp*.* PCC 6803

**DOI:** 10.1242/bio.017129

**Published:** 2016-03-30

**Authors:** Helena Čelešnik, Anja Tanšek, Aneja Tahirović, Angelika Vižintin, Jernej Mustar, Vita Vidmar, Marko Dolinar

**Affiliations:** Chair of Biochemistry, Faculty of Chemistry and Chemical Technology, University of Ljubljana, Ljubljana 1000, Slovenia

**Keywords:** *Synechocystis*, Cyanobacteria, Biocontainment, Biosafety, Nuclease *nucA*, Toxin/antitoxin

## Abstract

In recent years, photosynthetic autotrophic cyanobacteria have attracted interest for biotechnological applications for sustainable production of valuable metabolites. Although biosafety issues can have a great impact on public acceptance of cyanobacterial biotechnology, biosafety of genetically modified cyanobacteria has remained largely unexplored. We set out to incorporate biocontainment systems in the model cyanobacterium *Synechocystis* sp. PCC 6803. Plasmid-encoded safeguards were constructed using the nonspecific nuclease NucA from *Anabaena* combined with different metal-ion inducible promoters. In this manner, conditional lethality was dependent on intracellular DNA degradation for regulated autokilling as well as preclusion of horizontal gene transfer. In cells carrying the suicide switch comprising the *nucA* gene fused to a variant of the *copM* promoter, efficient inducible autokilling was elicited. Parallel to nuclease-based safeguards, cyanobacterial toxin/antitoxin (TA) modules were examined in biosafety switches. Rewiring of *Synechocystis* TA pairs *ssr1114/slr0664* and *slr6101/slr6100* for conditional lethality using metal-ion responsive promoters resulted in reduced growth, rather than cell killing, suggesting cells could cope with elevated toxin levels. Overall, promoter properties and translation efficiency influenced the efficacy of biocontainment systems. Several metal-ion promoters were tested in the context of safeguards, and selected promoters, including a *nrsB* variant, were characterized by beta-galactosidase reporter assay.

## Abstract (precis)

**Summary:** Biosafety of biotechnologically important microalgae was addressed by suicide switch implementation in cyanobacterium *Synechocystis* sp*.* PCC 6803. This is the first report of biocontainment safeguards in cyanobacteria.

## INTRODUCTION

Cyanobacteria display high metabolic versatility and are acquiring interest for biotechnological applications, as they are photosynthetic autotrophs that require little more than water, sunlight and CO_2_ for their growth and sustainable production of valuable metabolites. Engineered cyanobacteria can be used for generating a number of metabolites ranging from nutrients for human consumption to bioplastics and biofuels (Lai and Lan, 2015; Lau et al., 2015). Even though biosafety issues can have a fundamental impact on public opinion and acceptance of cyanobacterial biotechnology, biosafety of genetically modified cyanobacteria has remained largely unexplored. While modified cyanobacteria can be efficiently contained within closed photobioreactors, there is always a risk of accidental release, and uncertainty about possible negative ecological effects in case the engineered bacteria survive and establish themselves in the environment. Further risk is presented by cultivation of cyanobacteria in open reactors, raceways and ponds. Cyanobacteria have also been studied for bioremediation (Lau et al., 2015; Pereira et al., 2011), which would require monitoring of their persistence in the environment. The possibility of horizontal transfer of recombinant genetic material from genetically modified cyanobacteria to endogenous population is additionally concerning, considering cyanobacteria are widely present in nature (Li et al., 2001; Vioque, 2007) and some strains are naturally transformable (Vioque, 2007).

To reduce the likelihood of environmental persistence of engineered organisms, they can be equipped with biological containment mechanisms. In passive containment systems, specific gene defects can be engineered to make cell viability dependent on addition of exogenous supplement. In active containment systems, cells can be equipped with safeguards or ‘kill switches’ that can cause cell death in a controlled suicide process. Such microbes are intended to survive normally in the absence of kill switch induction, but in the environment, engineered cells can be destroyed by induced lethality, while leaving the surrounding endogenous microbes intact. Different lethal genes have been employed in conditional cell-killing systems in various bacteria. For example, cell death has been achieved by proteins that destroy the cell membrane (Bej et al., 1988; Schweder et al., 1992), the cell wall (Morita et al., 2001), that degrade RNA (Munthali et al., 1996; Torres et al., 2003; Wright et al., 2015), or destroy cellular DNA (Ahrenholtz et al., 1994; Balan and Schenberg, 2005; Torres et al., 2003). The genes encoding these toxic proteins have been put under the control of different types of promoters, such as those inducible by increased temperature (Ahrenholtz et al., 1994), starvation (Schweder et al., 1992), the presence of specific chemicals (Bej et al., 1988; Jensen et al., 1993; Munthali et al., 1996) or growth supplements (Balan and Schenberg, 2005; Schweder et al., 1992). Employing more than one containment system in a single cell has been shown to increase the efficacy of biological safeguards (Torres et al., 2003).

While biological containment systems have been implemented in various organisms (Moe-Behrens et al., 2013; Wright et al., 2013), none have so far been developed for cyanobacteria. Even though induced lysis has been carried out in cyanobacteria for the purpose of recovering biofuel from biomass (Liu and Curtiss, 2009; Miyake et al., 2014), metabolite extraction does not require complete lysis-induced death, while total killing is necessary for biosafety purposes.

In this work, we addressed biosafety of genetically modified cyanobacteria through construction of biological containment systems in the model cyanobacterium *Synechocystis* sp. PCC 6803 (hereafter *Synechocystis*). Two strategies were employed. In the first, conditional lethality was dependent on intracellular degradation of DNA, to accomplish not only regulated killing of cells but also destruction of cellular genetic material, which could otherwise be available for horizontal gene transfer. To that end, we made use of non-specific DNA/RNA nuclease NucA and its inhibitor NuiA from the cyanobacterium *Anabaena* sp. PCC 7120. By using metal-ion inducible promoters to trigger nuclease expression, we were able to elicit efficient cell killing upon inducer addition. The most efficient promoter was a P*copM* variant. In the second approach, *Synechocystis* toxin-antitoxin (TA) systems *ssr1114/slr0664* and *slr6101/slr6100* were rewired for conditional lethality by using metal-ion inducible promoters. In different kill switch variants with toxins Slr0664 or Slr6100 (which encode RelE-like ribonucleases), reduced growth of bacteria rather than efficient cell killing was observed, suggesting bacteria were able to cope with the cellular damage inflicted by the toxins. Finally, as the choice of promoters used in cyanobacterial conditional suicide systems was crucial, several metal-ion promoters were tested in the context of kill switches, and selected promoters were characterized in detail by beta-galactosidase reporter assay.

## RESULTS

### Nuclease-based cyanobacterial kill switch

**Fig. 1.**
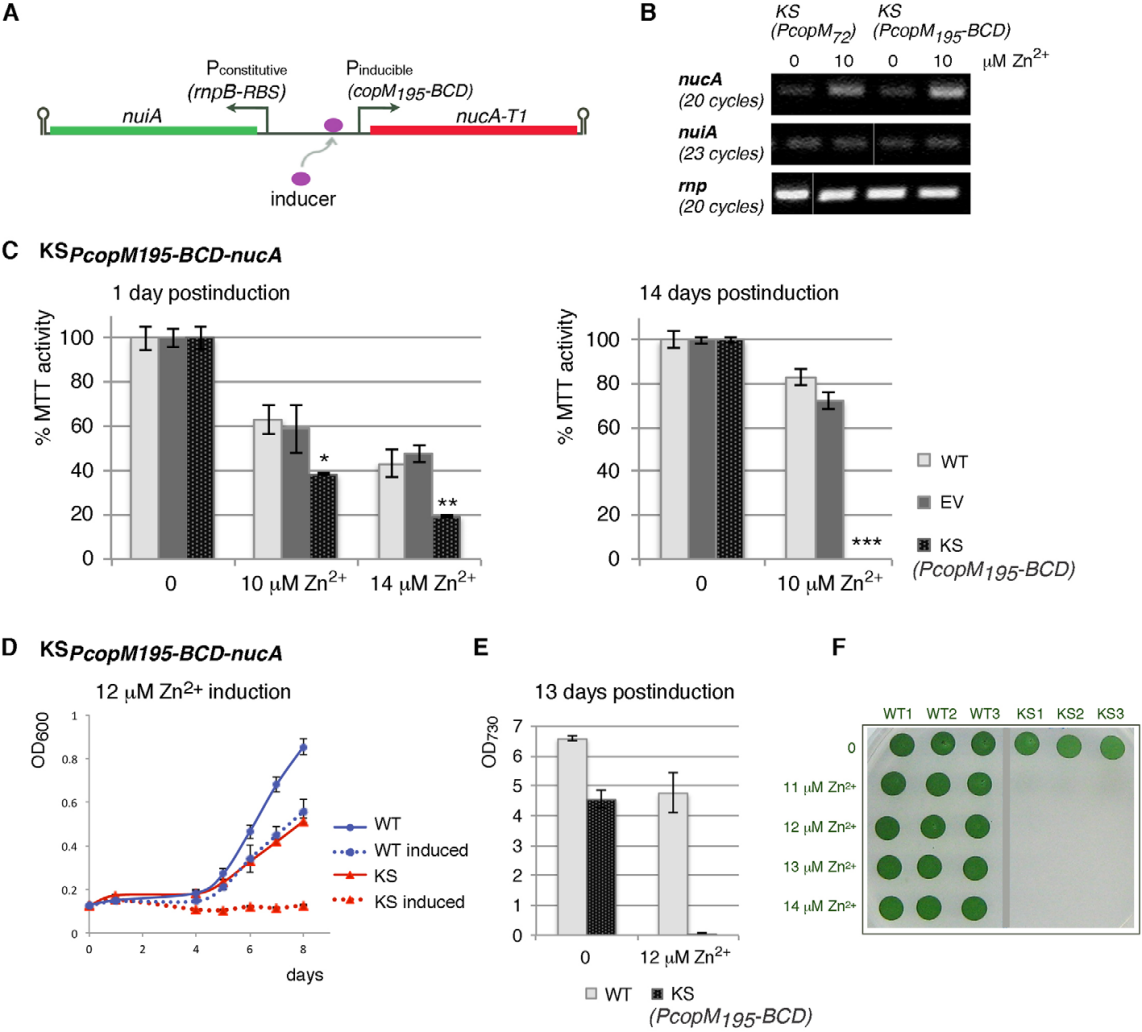
***Synechocystis* sp*.* PCC 6803 carrying the plasmid-encoded nuclease suicide switch KS*_PcopM__195-BCD__-nucA_* displays efficient induced autokilling.** (A) Diagrammatical representation of the suicide switch. The nuclease gene is under the inducible promoter P*copM_195_-BCD* (P_inducible_) that enables triggering of cell death upon exposure to inducer. To protect cells from possible leaky nuclease expression in the absence of inducer, the nuclease inhibitor is produced from the weak constitutive promoter P*rnpB-RBS* (P_constitutive_). *T1*, degradation tag; arrow indicates transcriptional start site; hairpin indicates transcription terminator. (B) RT-PCR analysis of *nucA* and *nuiA* mRNA levels in kill switch *Synechocystis* cells KS*_PcopM72-nucA_* and KS*_PcopM195-BCD-nucA_* 1 h after 10 µM Zn^2+^ induction. For detection of the less abundant *nuiA*, more PCR cycles were needed. *rnp*, loading control. (C) MTT assay showing a drop in viability of suicide switch *Synechocystis* cells KS*_PcopM195-BCD-nucA_* (KS) 1 day or 14 days after induction with Zn^2+^ (mean % MTT activity±s.e.m., *n*=3). WT, wild type; EV, empty vector control. Asterisks indicate significant differences (KS compared with WT) at *(1 day, 10 µM Zn^2+^); **(1 day, 14 µM Zn^2+^); ***(14 days, 10 µM Zn^2+^) (*t*-test, *P*<0.05). Differences between WT and EV were not statistically significant. (D) Growth of wild-type *Synechocystis* (WT) and KS*_PcopM195-BCD-nucA_* (KS) cells was measured by plate reader after induction with 12 µM Zn^2+^ (mean OD_600_±s.e.m.). (E) Cell density **(**OD_730_) of cultures in multiwell plates from (D) was determined manually on the last day of growth (OD_730_±s.e.m.). By assessing culture density at OD_730_ at the end of multiwell plate experiment, we validated that the efficiency of killing determined by OD_600_ (plate reader) and OD_730_ were comparable. Kill switch KS*_PcopM195-BCD-nucA_* cells showed complete killing upon 12 µM Zn^2+^ induction. (F) Triplicate aliquots from cultures in (D) were plated on BG11 solid media at day 13. Complete killing was observed for KS*_PcopM195-BCD-nucA_* cells (triplicates KS1-3) at or above 12 µM Zn^2+^, while some survivors were seen at 11 µM Zn^2+^. WT1-3, wild-type triplicates.

In order to construct biosafety mechanisms in cyanobacteria, we took advantage of the cyanobacterial non-specific DNA/RNA nuclease NucA and its inhibitor NuiA from *Anabaena*. While *Synechocystis* sp*.* PCC 6803 does not contain a NucA homolog, nucleases of this type are present in several bacterial species and are believed to have evolved to serve for nutritional purposes and sometimes as bacteriocides (Meiss et al., 1998; Muro-Pastor et al., 1992). We envisioned that by rewiring the nuclease/inhibitor pair for conditional expression, cell survival could be achieved specifically in the photobioreactor, while upon accidental release into the environment, the rewired nuclease would prevail over the inhibitor, thereby killing the cells. To create such a mechanism, the nuclease gene was placed under an inducible promoter to allow induction upon exposure to environmental inducer (Fig. 1A). The coding sequence of *nucA* was shortened by 69 nucleotides encoding the signal peptide (Muro-Pastor et al., 1992) in order to achieve intracellular localization of the nuclease by preventing its export to the periplasm. To protect cells from possible leaky nuclease production in the bioreactor in absence of inducer, the nuclease inhibitor gene was fused to a weak constitutive promoter (Fig. 1A).

### Genetic elements used in suicide switch construction

The choice of promoters was crucial for creating a successful suicide mechanism. In particular, for the fusion with the toxic nuclease, we expected that low leakiness and high promoter inducibility would be needed, with the former necessary to preclude any negative effects on growth in absence of inducer. For potential future biotechnological use, the cost of promoter inducer was also a factor. Even though numerous tight and highly responsive promoters are well characterized in *Escherichia coli* (e.g. P*trc*, P*lac*), these promoters do not work comparably in cyanobacteria, likely due to RNA polymerase differences (Heidorn et al., 2011). Some have been shown to lose their inducer responsiveness in cyanobacteria and become constitutive (Guerrero et al., 2012; Heidorn et al., 2011). Current cyanobacterial molecular biology toolbox offers only a limited number of well-characterized promoters to carry out expression of a gene of interest in *Synechocystis*. Several of the most inducible and tightly regulated cyanobacterial promoters belong to the group of native metal-ion responsive promoters. Examples include promoters preceding two operons involved in copper response, the *copBAC* operon (Giner-Lamia et al., 2012) and the *copMRS* operon (Giner-Lamia et al., 2015, 2012), the nickel-response operon *nrsBACD* (Blasi et al., 2012; Lopez-Maury et al., 2002; Peca et al., 2008), the metallothionein gene *smtA* (Turner et al., 1996), the plastocyanin gene *petE* (Briggs et al., 1990), the cytochrome c6 gene *petJ* (Kuchmina et al., 2012) and the *coaT* gene (Guerrero et al., 2012; Peca et al., 2008). These promoters react to very low (micromolar) concentrations of metal ions and typically respond to several metal ions, showing variation in response depending on the metal ion used. As inducers, metal ions are cost-efficient for larger culture volumes. For construction of inducible biosafety circuits in cyanobacteria, we selected three metal-ion responsive promoters: P*copM*, P*copB,* and P*nrsB* (Table 1).

**Table 1.**
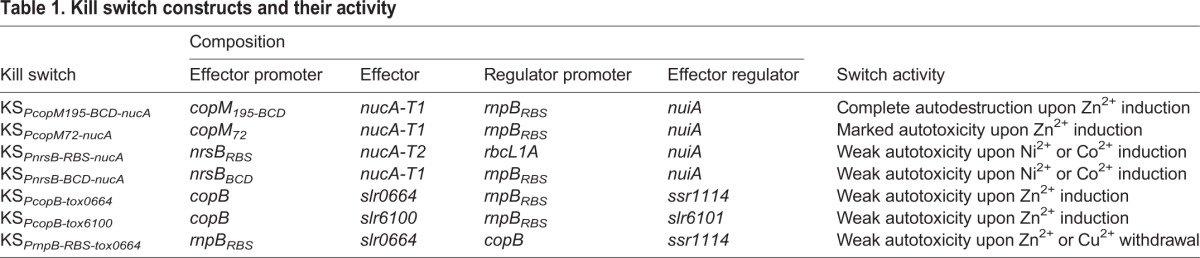
**Kill switch constructs and their activity**

Initial attempts at cloning the suicide switch by trying to use different metal-ion responsive promoters to drive nuclease expression were repeatedly unsuccessful. Either no transformants were obtained or nuclease-disabling mutations were observed, probably due to leaky expression and cellular toxicity of the nuclease. Cloning was feasible only when degradation tags (specific amino acid sequences recognized by cytoplasmic proteases) were used to curtail the cellular half-life of the nuclease. Tags T1 (RPAANDENYAAAV) or T2 (RPAANDENYALAA) (Huang et al., 2010; Landry et al., 2013) were cloned as C-terminal fusions to the nuclease. T1 has been shown to cause weaker reporter protein destabilization in cyanobacteria than T2 (Huang et al., 2010; Landry et al., 2013).

### Native P*copM* promoter in nuclease-based suicide switch

We first constructed a plasmid-encoded nuclease-based kill switch (KS*_PcopM72-nucA_*) in which *nucA* was fused to promoter P*copM* (Fig. S1A; Table 1). In *Synechocystis*, this promoter regulates the *copMRS* operon, which is involved in copper response (Giner-Lamia et al., 2015). Under native conditions, P*copM* responds robustly to Cu^2+^. As little as 1 µM Cu^2+^ triggers about 26-fold increase in CopM protein levels, and 3 µM Cu^2+^ increases *copM* mRNA levels 75-fold (Giner-Lamia et al., 2015, 2012). Other metal ions (Zn^2+^, Cd^2+^, and Ni^2+^) trigger a poorer P*copM* response (Giner-Lamia et al., 2012). The coding sequence of *nucA* (with T1 degradation tag) was fused to the 72-nt long P*copM_72_* promoter sequence up to the start codon, containing two direct repeats that are likely involved in transcriptional regulation (Giner-Lamia et al., 2012). The *nuiA* gene (encoding the inhibitor) was feebly expressed (Fig. 1B) from the weak constitutive promoter P*rnpB* (promoter of ribozyme RNase P subunit B) fused to a ribosome binding site (RBS) (Huang et al., 2010). After induction of *Synechocystis KS_PcopM72-nucA_* culture (10^6^ cells/ml) with 2 µM Cu^2+^, no significant killing was observed (Fig. S1B). Rather, slower growth was recorded in comparison to wild-type (WT) cells, as measured by optical density (OD_730_). Some reduced growth was also seen with uninduced KS*_PcopM72-nucA_* cells, possibly due to the use of standard BG11 growth medium, which already contains small amounts of Cu^2+^ (0.32 μM) (Rippka, 1988). However, the choice of this medium was intentional in order to mimic conditions of bioreactor cultivation for biotechnological purposes.

Although metal ions are required for essential cellular processes, they become cytotoxic after exceeding specific critical concentrations. *Synechocystis* has a rather low tolerance for Cu^2+^ (Blasi et al., 2012) and in our experiments, substantial general toxicity toward WT *Synechocystis* cells is observed at Cu^2+^-ion concentrations above 4 µM, thereby limiting usefulness of Cu^2+^ as promoter inducer. On the other hand, *Synechocystis* tolerance to Zn^2+^ is relatively high, with IC_50_ (half growth inhibitory concentration) between 8 and 16 µM Zn^2+^ (Blasi et al., 2012). When KS*_PcopM72-nucA_* culture was induced with 10 µM Zn^2+^, a substantial, although incomplete killing of suicide switch cells was observed in comparison to WT cells, which retained normal growth (Fig. S1B). Moreover, uninduced KS*_PcopM72-nucA_* cells did not display significant growth inhibition in spite of the presence of 0.77 μM Zn^2+^ (Rippka, 1988) in standard BG11 medium.

### Bicistronic design in P*copM*-nuclease-based suicide switch

In order to improve efficiency of this suicide system, two construct modifications were made (Fig. S1C). First, a larger sequence segment (195 nt) preceding the *copMRS* operon was included in the suicide switch to increase the likelihood that all the important regulatory sequences were contained in the promoter fragment. Second, 26 nucleotides of the native sequence (from the *copM* transcriptional start site to the start codon) were replaced by bicistronic design (BCD) (Mutalik et al., 2013) (Fig. S1C). BCD is a genetic element that standardizes the mRNA secondary structure over the RBS–CDS (ribosome binding site – coding sequence) junction, thereby diminishing variation in ribosome binding and expression when combined with different genes of interest. It allows reliable initiation of translation of random genes (Mutalik et al., 2013). With the resulting modified construct (KS*_PcopM195-BCD-nucA_*), complete killing of *Synechocystis* cells was achieved upon addition of Zn^2+^ ions, as expected for a functional kill switch (Fig. 1, Table 1).

First, induction of KS*_PcopM195-BCD-nucA_* cells with 10 µM Zn^2+^ markedly increased *nucA* transcript levels within an hour of induction (comparably to the increase seen in KS*_PcopM72-nucA_* cells) (Fig. 1B). This correlated with a sharp decrease in the number of viable, metabolically active cells, as quantified by the MTT assay. This assay measures the conversion of MTT [3-(4,5-dimethylthiazol-2-yl)-2,5-diphenyl tetrazolium bromide] into formazan crystals by active reductases in living cells (Li and Song, 2007) (Fig. 1C). Within a day of adding inducer to log-phase cultures, MTT-reducing activity of KS*_PcopM195-BCD-nucA_* culture significantly decreased (*P*<0.05 compared with WT) to 38%±0.7% at 10 µM Zn^2+^ and 19%±0.7% at 14 µM Zn^2+^. Activity of WT cells and cells carrying an empty vector (EV) also decreased (to 63%±6.7% (WT) and 59%±11.1% (EV) at 10 µM Zn^2+^; and to 43%±6.4% (WT) and 47%±3.8% (EV) at 14 µM Zn^2+^), reflecting general toxic effects of zinc ions. However, over a 14-day period WT and EV cultures were mostly able to recover, while KS*_PcopM195-BCD-nucA_* cells completely lost ability to reduce MTT, indicating they were no longer viable (Fig. 1C). Growth curves corroborated MTT results. When bacterial cultures (10^6^ cells/ml) were induced with 12 µM Zn^2+^, the suicide switch culture showed no growth even after prolonged incubation (Fig. 1D,E). Plating of this culture on solid media at day 13 revealed no survivors (Fig. 1F). In contrast, WT cells remained viable, albeit displaying a small retardation of growth (Fig. 1D). Some growth hindrance was seen for suicide switch cells without Zn^2+^-induction (Fig. 1D), reflecting weak leakiness of nuclease expression (Fig. 1B). Together, these results indicate that the kill switch KS*_PcopM195-BCD-nucA_* is functional.

As noted, the kill switch KS*_PcopM195-BCD-nucA_* differed from the kill switch KS*_PcopM72-nucA_* twofold: in the length of the P*copM*-promoter fragment, important for *nucA* transcription, and in the use of the BCD element (versus the native RBS), important for translation initiation. Induction of these two switches resulted in different killing efficiencies (Fig. 1D, Fig. S1B), even though upregulation of *nucA* transcript levels was comparable for both kill switch cells (Fig. 1B). This suggests that translation initiation efficiency (influenced by BCD) contributed to the improved killing observed for KS*_PcopM195-BCD-nucA_* cells.

### Determining optimal inducer concentration for autodestruction

Up to 11 µM Zn^2+^, survivors of induced KS*_PcopM195-BCD-nucA_* cells could readily be observed. Complete killing was typically achieved at concentrations of 12 to 14 µM Zn^2+^ and culture density of 10^6^ cells/ml (Fig. 1E). Concentrations above 14 µM Zn^2+^ were not useful due to nonselective Zn^2+^-toxicity to WT cells. Nonetheless, at higher culture density of suicide switch cells, the killing efficiency with 12 to 14 µM Zn^2+^ was reduced, suggesting that inducer may not have reached all cells or that it may have been getting removed from the cells. In agreement with this is our observation that WT cells also respond variably to Zn^2+^ at different culture densities (data not shown). Therefore, for real-life biotechnological applications, attention to inducer concentration as well as cell density will be necessary. Notably, an occasional biological replicate of KS*_PcopM195-BCD-nucA_* cells started growing even at 12-14 µM Zn^2+^ at 10^6^ cells/ml. It is possible that selective pressure imposed by induced nuclease expression may have led to suicide switch instability.

### Promoter P*nrsB* in nuclease-based suicide device

**Fig. 2.**
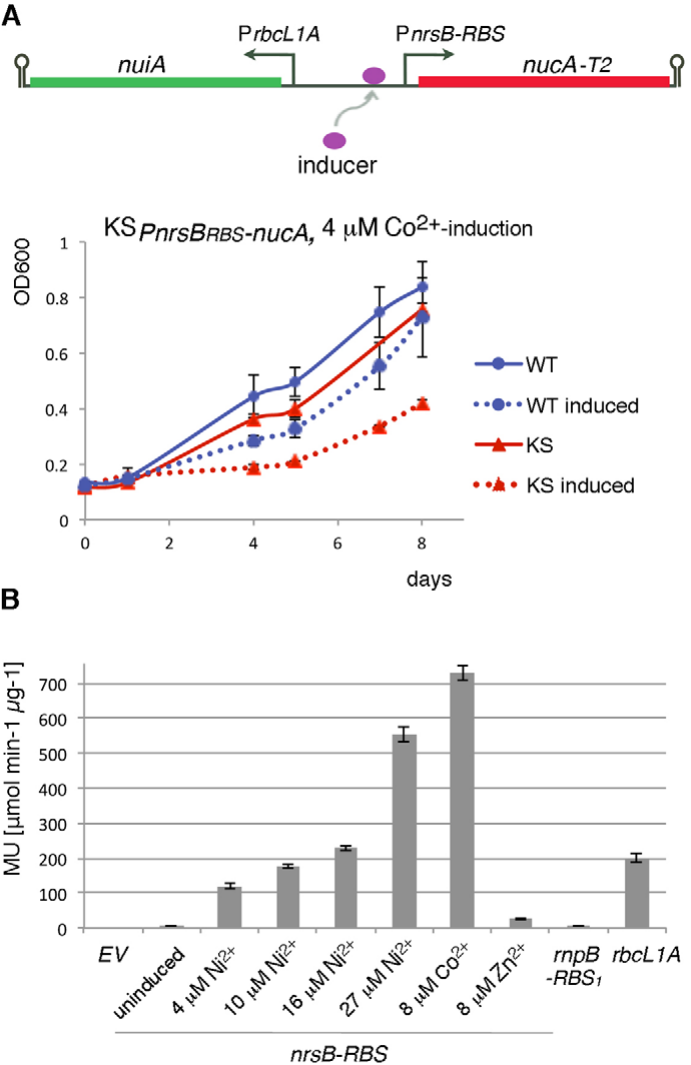
**Characterization of P*nrsB-RBS* promoter element in *Synechocystis* and its use in the nuclease-based suicide switch KS*_PnrsB__RBS__-nucA_*.** (A) Diagrammatical representation of the KS*_PnrsBRBS-nucA_* suicide switch (top panel). Nuclease expression is driven by the inducible promoter P*nrsB-RBS* and expression of nuclease inhibitor by the moderate constitutive promoter P*rbcL1A*. *T2*, degradation tag; ; arrow indicates transcriptional start site; hairpin indicates transcription terminator. (Bottom panel) Growth of wild-type (WT) and KS*_PnrsBRBS-nucA_* (KS) *Synechocystis* cells was recorded by a plate reader after induction with 4 µM Co^2+^ (mean OD_600_±s.d.) (B) Evaluation of promoter activity by beta-galactosidase reporter analysis, represented as mean standardized MU (μmol ONP min^−1^ μg^−1^)±s.e.m. Promoter P*nrsB-RBS* (induced with different metal ions) and constitutive promoters P*rnpB-RBS_1_* and P*rbcL1A* were analyzed. EV, empty vector control.

We also assessed the P*nrsB* promoter, which regulates the expression of the *nrsBACD* operon involved in cellular response to Ni^2+^ (Garcia-Dominguez et al., 2000; Lopez-Maury et al., 2002). P*nrsB* is induced by Ni^2+^ and Co^2+^ ions and exhibits low leakiness (Garcia-Dominguez et al., 2000; Lopez-Maury et al., 2002). The BG11 growth medium does not include Ni^2+^, but it contains 0.17 μM Co^2+^ (Rippka, 1988). To construct the suicide switch, *nucA-T2* (gene encoding NucA with T2 degradation tag) was fused to the sequence P*nrsB-RBS*, which is an intergenic segment preceding the *nrsB* gene*,* in which a strong RBS (Heidorn et al., 2011) replaced the native RBS (Table 1; Fig. S2A,B). The strong RBS was used to improve chances of strong nuclease expression. The nuclease inhibitor in the switch was moderately expressed from the constitutive *rbcL1A* promoter (Fig. 2A) (Huang et al., 2010). The resulting construct (KS*_PnrsBRBS-nucA_*) caused only moderate autotoxicity rather than efficient killing when induced with 4 µM Co^2+^ (Fig. 2A), precluding its usefulness for biotechnological purposes. At Co^2+^ concentrations higher than 4 µM, strong general toxicity to WT cells was observed, in line with the published IC_50_ of 8 µM for Co^2+^ (Blasi et al., 2012).

Interestingly, although P*nrsB* has been identified as a Ni^2+^-promoter that responds most robustly to Ni^2+^ ions, in the suicide switch context cobalt-triggered autotoxicity measured by the MTT assay exceeded that observed with Ni^2+^ induction (Fig. S2D,E), even though 4 µM Ni^2+^ did markedly induce *nucA* mRNA levels in KS*_PnrsBRBS-nucA_* cells (Fig. S2D). To understand the characteristics of the P*nrsB-RBS* genetic element, we set out to analyze it using the beta-galactosidase reporter assay (Miller, 1972). In agreement with the kill switch observations, P*nrsB-RBS* fused to *lacZ* responded well to both ions, with a more pronounced reaction to Co^2+^ than Ni^2+^ (Fig. 2B). At 8 µM Co^2+^, a 105-fold induction was observed, significantly higher than that with 10 µM Ni^2+^ (26-fold), or even 27 µM Ni^2+^ (80-fold promoter induction, accompanied by strong general toxicity to cells). Such inducer specificity differs from that of native P*nrsB* (Garcia-Dominguez et al., 2000; Lopez-Maury et al., 2002; Peca et al., 2007). It is not clear what causes the difference in specificity. It is possible that the difference may reflect sequence modifications in the RBS region (e.g. perhaps causing secondary structure changes). In the P*nrsB-RBS* fusion constructs, the sequence of the *nrsB*-regulator (*nrsR*) and sensor (*nrsS*) (Lopez-Maury et al., 2002) were not included (promoter response relied on endogenous NrsR and NrsS), which also may have influenced promoter activity.

Although transcriptional induction of KS*_PnrsBRBS-nucA_* was pronounced (Fig. S2D), autotoxicity was not efficient, possibly due to issues with translation. To optimize chances of efficient translation initiation, bicistronic design (BCD) was added to the promoter sequence to create P*nrsB-BCD* (Fig. S2C). Nonetheless, inclusion of bicistronic design did not improve killing efficiency, as tested with a kill switch, in which *nucA-T1* was expressed from P*nrsB-BCD* and *nuiA* from weak P*rnpB* promoter (Table 1). Therefore, other factors may have played a role in low kill switch efficiency.

### Toxin/antitoxin-based cyanobacterial kill switch

In the second approach, molecular safeguards were created in *Synechocystis* sp. PCC 6803 by making use of cyanobacterial toxin-antitoxin (TA) systems. TAs are widespread prokaryotic genetic elements comprising a stable toxic protein and a less stable neutralizing antidote involved in various cellular functions ranging from plasmid addiction to stress response (Otsuka, 2016). Two *Synechocystis* TA systems were employed in safeguard constructs: the chromosomally encoded pair *ssr1114/slr0664*, for which lethal toxicity had been shown in the heterologous host *E. coli* (Ning et al., 2011; Ye and Ning, 2010); and the plasmid-encoded putative TA pair *slr6101/slr6100*, which was selected based on bioinformatics data (Makarova et al., 2009) (TADB database, last accessed January 15, 2016, http://202.120.12.135/TADB2/index.php) and our preliminary toxicity evaluation in *E. coli* (unpublished data). Both toxin genes (*slr0664* and *slr6100*) encode proteins with homology to RNA interferase RelE. Similar to nuclease-based kill switch design, we employed metal-ion inducible promoters to rewire *Synechocystis* TA pairs for conditional expression.

### Use of *cop* promoters in toxin/antitoxin-based kill switch

We first attempted to construct a TA-based kill switch by using the *copM* promoter, which proved optimal in the nuclease-based switch. However, our repeated cloning efforts resulted in constructs with toxin-disabling mutations, suggesting occurrence of promoter leakage and selection pressure.

**Fig. 3.**
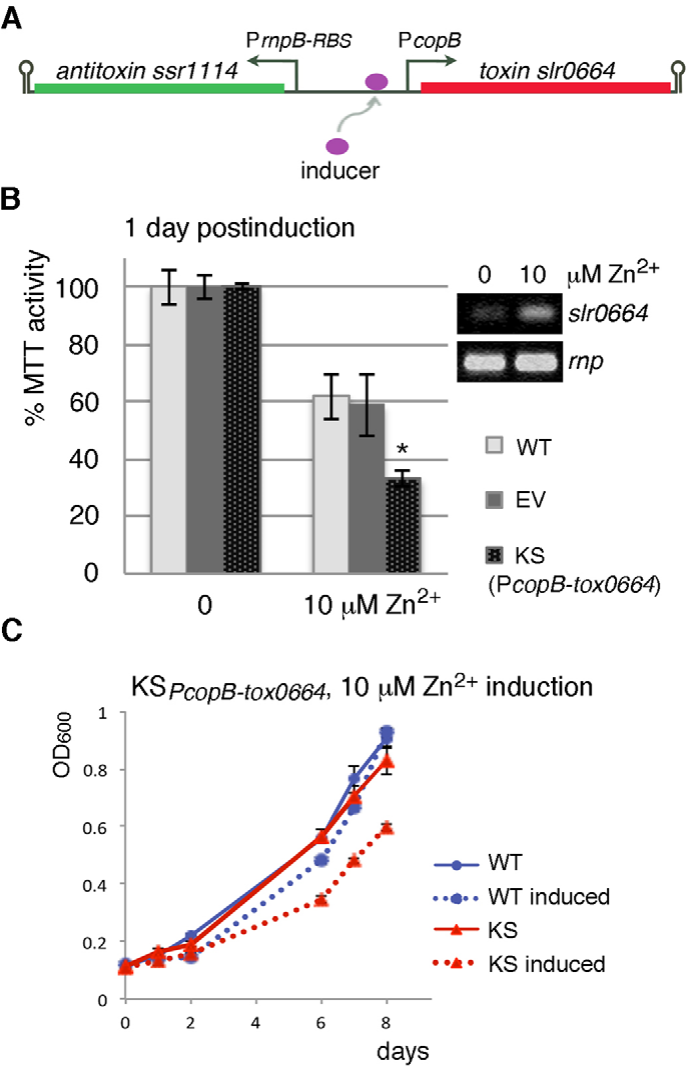
**Induced autotoxicity of *Synechocystis* sp. PCC 6803 cells carrying the plasmid-encoded toxin/antitoxin suicide switch KS*_PcopB-tox0664_*.** (A) Diagrammatical representation of the KS*_PcopB-tox0664_* suicide switch*.* Toxin expression is driven by the inducible promoter P*copB* and expression of antitoxin by the weak constitutive promoter P*rnpB-RBS.* Arrow indicates transcriptional start site; hairpin indicates transcription terminator. (B) MTT assay showing a drop in viability of suicide switch *Synechocystis* cells KS*_PcopB-tox0664_* (KS) 1 day after induction with 10 µM Zn^2+^ (mean % MTT activity±s.e.m., *n*=3). WT, wild type; EV, empty vector control. Asterisk indicates significant difference at 10 µM Zn^2+^ (KS compared with WT, *t*-test, *P*<0.05). Differences between WT and EV were not statistically significant. (Inset) Increase in toxin mRNA levels is detected by RT-PCR in *Synechocystis* KS*_PcopB-tox0664_* cells 1 h after induction with 10 µM Zn^2+^; *rnp*: loading control. (C) Growth of wild-type *Synechocystis* (WT) and KS*_PcopB-tox0664_* (KS) cells was recorded by a plate reader after induction with 10 µM Zn^2+^ (mean OD_600_±s.e.m., *n*=3).

Construction was successful with P*copB*, another promoter involved in cellular response to copper (Giner-Lamia et al., 2012). It responds strongly to Cu^2+^, with 14-fold induction at 3 µM Cu^2+^, moderately to Zn^2+^, and is fairly tight when uninduced (Giner-Lamia et al., 2012). Due to higher *Synechocystis* tolerance to Zn^2+^, this metal ion was used for induction. Kill switches containing the toxin (either *slr0664* or *slr6100*) fused to *copB* (Fig. S3), with antitoxins (*ssr1114* or *slr6101,* respectively) expressed from the weak *PrnpB-RBS* triggered cellular autotoxicity (Fig. 3, Table 1). Nevertheless, neither suicide switch caused efficient killing. Due to similar performances, only *slr0664* kill switch KS*_PcopB-tox0664_* is shown (Fig. 3).

A day after induction of log-phase KS*_PcopB-tox0664_* culture with 10 µM Zn^2+^, MTT assay revealed a significant, 77%±3.0% drop in metabolically viable cells (Fig. 3B), which is comparable to that seen with the efficient nuclease switch KS*_PcopM195-BCD-nucA_* (Fig. 1C). The drop correlated with increased toxin *slr0664* mRNA levels (Fig. 3B). However, following induction, the cells carrying the toxin kill switch continued to grow with reduced rate (Fig. 3C), unlike the nuclease cells KS*_PcopM195-BCD-nucA_* that died off (Fig. 1D). One explanation for different responses of the nuclease and the toxin safeguards may be that the damage inflicted by the toxin ribonuclease activity may be easier for cells to cope with than the damage imposed by NucA, which is DNA and RNA nuclease. It is also possible that the induced toxin protein levels in the kill switch cells were low (e.g. due to translation and degradation rates). One explanation may be the existence of endogenous Ssr1114 antitoxin in *Synechocystis*, which may be neutralizing the induced toxin. As antitoxin crosstalk has been described (Smith et al., 2012), it is also conceivable that other endogenous antitoxins may be suppressing the toxin. Consequently, in order for TA-based kill switch to be efficient, the toxin levels would have to be heavily induced to inflict substantial cellular damage as well as override possible neutralizing molecules. Toxin induction with promoter P*copB* does not appear to have reached that intensity.

### TA-safeguard with antitoxin induction during growth in culture medium

Another possible safeguard mechanism involves keeping both the toxin and the antitoxin expressed in the bioreactor, with the antitoxin induced by inducer that is scarce in the environment, and the toxin being continually expressed. Consequently, in the environment, the toxin is expected to prevail over the antitoxin. Potentially good inducers for this purpose may be zinc ions, considering that publicly available data on metal ion content in riverine environments (e.g. for Slovenia: http://www.arso.gov.si) indicate that zinc concentrations tend to be low (around 0.08 µM).

**Fig. 4.**
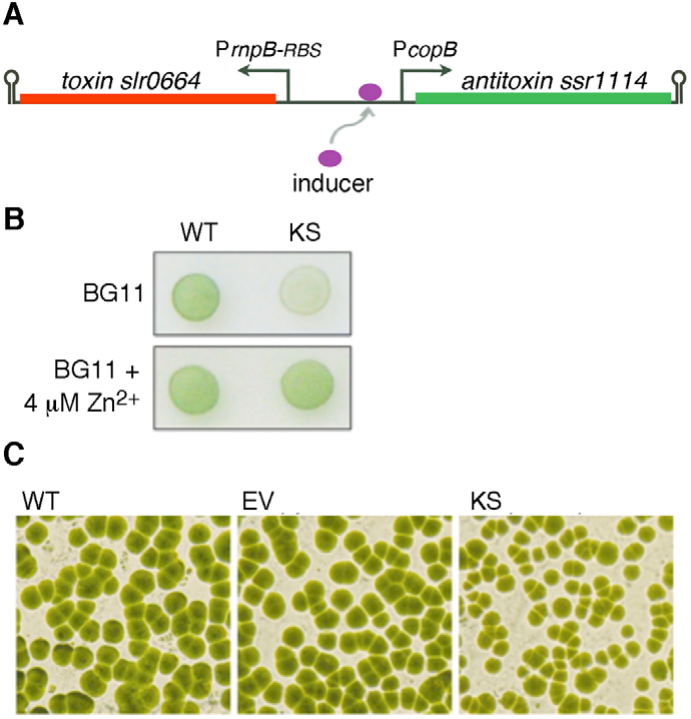
**Autotoxicity triggered by withdrawal of antitoxin inducer in *Synechocystis* sp. PCC 6803 cells carrying a plasmid-encoded toxin/antitoxin suicide switch.** (A) Diagrammatical representation of the suicide switch. Antitoxin expression is driven by the inducible promoter P*copB* and expression of toxin by the constitutive promoter P*rnpB-RBS.* Antitoxin levels can be reduced upon withdrawal of inducer from the growth medium, allowing the toxin to prevail. Arrow indicates transcriptional start site; hairpin indicates transcription terminator. (B) Growth of wild type *Synechocystis* (WT) and suicide switch cells (KS) on BG11 plates with or without added Zn^2+^. Inducer limitation in BG11 plates results in autotoxicity of suicide switch cells. (C) Stereomicroscopic evaluation of colonies from agar plates in (B) reveals that kill switch cells (KS) exhibit similar numbers of colony forming units but different growth rates in comparison to wild-type *Synechocystis* (WT) and empty vector (EV) control.

A kill switch in which the toxin *slr0664* was expressed constitutively from *PrnpB-RBS* and the antitoxin *ssr1114* was inducible from the P*copB* promoter (Fig. 4A, Table 1) was tested in *Synechocystis*. As expected, growth of these cells on BG11 plates containing 4 µM Zn^2+^ was indistinguishable from that of WT cells (Fig. 4B). In contrast, substantially less growth was observed for kill switch cells on BG11 alone. Plate colony counting and stereomicroscopic evaluation revealed that toxin expression resulted in cell growth inhibition rather than cell killing, as indicated by a marked change in colony size but similar colony counts between plates (Fig. 4C). This suggests that kill switch cells are able to cope with the damage imposed by toxin activity, and higher toxin levels may be necessary to achieve efficient killing. Thus, in order for this safeguard mechanism to be useful for biotechnological purposes, other promoter combinations would have to be tested.

## DISCUSSION

In this work, a functional biocontainment mechanism was implemented in the model cyanobacterium *Synechocystis* sp. PCC 6803. Our biosafety suicide switch KS*_PcopM195-BCD-nucA_* was based on induction of an intracellular DNA/RNA nuclease NucA, which causes killing of cells by degrading their genetic material. The nuclease gene was driven by a variant of the metal-ion inducible promoter *copM* (P*copM_195_-BCD*). The advantage of having a nuclease incorporated into a biosafety system is the destruction of recombinant DNA, so that it cannot be available for horizontal transfer. As expected for a functional suicide switch, induction of nuclease expression in KS*_PcopM195-BCD-nucA_* cells resulted in efficient programmed autokilling. This is the first report of biocontainment implementation in *Synechocystis*. Being a standalone circuit, it remains to be tested if this biosafety device may be transferrable to other cyanobacteria. This system can serve as a starting point for further cyanobacterial biosafety research. The biocontainment safeguard was developed under constant laboratory growth conditions. For biotechnological applications, it will need further assessment under different conditions, for example fluctuating temperatures and light intensities. Additionally, local environmental inducer levels will have to be determined.

It should be noted that inducible lysis in *Synechocystis* has been carried out before; for the purpose of metabolite extraction, lysis has been triggered by bacteriophage-derived lysis genes (Liu and Curtiss, 2009; Miyake et al., 2014). However, for metabolite recovery, complete lysis-induced killing is not necessary, while it is required for biosafety purposes. Thus, for recovery of biofuel-related compounds from *Synechocystis*, partial cell lysis was observed with green-light inducible lytic system, with about 40% cell death recorded (Miyake et al., 2014). In another metabolite-recovery lysis system (Liu and Curtiss, 2009), the use of *nrsB* promoter caused efficient killing in *Synechocystis.* Nonetheless, the two most efficient strains with bacteriophage lysis genes showed substantial growth retardation, and the cells of these strains adhered to each other and to the container walls in absence of induction, indicating probable cell wall damage caused by likely leakage of lysis enzymes (Liu and Curtiss, 2009). These traits can be disadvantageous in biotechnological applications. The benefit of using a nuclease as the toxic protein in biosafety devices is avoiding such problems of adherence in absence of inducer.

To construct a cyanobacterial biosafety device, we tested different promoters (seeking good promoter inducibility and low promoter leakiness) and translation initiation elements. The efficient KS*_PcopM195-BCD-nucA_* suicide switch was regulated by P*copM_195_-BCD,* a variant of the metal-ion responsive promoter *copM*. P*copM_195_-BCD* in the kill switch showed good Zn^2+^-responsiveness. Additionally, putting a bicistronic translation initiation element BCD in place of the native RBS in the region preceding the *copM* start codon appears to have contributed to the killing efficiency of the switch. Nonetheless, the P*copM_195_-BCD* promoter displayed some leakiness, as determined by *nucA* transcript levels and a decrease in growth rate of uninduced kill switch cells in comparison to WT. This decrease suggested the expression level of the inhibitor in the kill switch may have been insufficient to completely neutralize the nuclease in absence of induction. Leakage can be a problem in suicide biosafety systems, as it causes reduced growth rate due to basal expression of the toxic gene; this can give selective growth advantage to cells with mutated suicide functions (Knudsen and Karlstrom, 1991). In agreement with this, sporadic biological replicates of kill switch KS*_PcopM195-BCD-nucA_* started growing in the presence of inducer. In addition to promoter leakiness and induction capability, the practical usefulness of kill switches can be influenced by the ability of the inducer to reach all cells and activate gene expression. At higher cell densities, reaching all cells may be a problem that can result in inconsistent induction. Furthermore, less inducer may be available per cell. Also possible in denser cultures are cell aggregation and biofilm formation that can pose a further challenge. Additionally, it has been reported that with varying cell density, promoter tightness can vary (described for a P*lac* promoter variant used in *Synechocystis*) (Guerrero et al., 2012). In denser cultures we have observed some variation in response of WT cells to the presence of metal ions, and some inconsistency in kill switch response to inducer. In biotechnological applications, all these limitations will need to be taken into consideration. Taking everything into account, we can envision that kill switch KS*_PcopM195-BCD-nucA_* is suitable to achieve a containment level where controllable reduction of genetically modified bacteria and decreased quantities of recombinant genetic material are desired, but complete elimination is not a requirement. When testing for a reduction or elimination of bacteria, it should be noted that available methods for monitoring cell survival cannot prove complete eradication of engineered strains, as there is always a possibility that the cells could be surviving in a dormant state.

Currently, cyanobacterial toolbox is lacking in really tight promoters with very high inducibility. Actually, cyanobacterial metal-ion responsive promoters are among the best available, even though their usefulness in biological safeguards is not ideal. Further work on cyanobacterial genetic elements will be required to improve our understanding of cyanobacterial promoters. We have contributed to the cyanobacterial toolbox by analyzing P*nrsB-RBS* (a *nrsB* promoter variant) with beta-galactosidase assay. Interestingly, unlike the native P*nrsB*, this variant exhibits better responsiveness to Co^2+^ than Ni^2+^ ions.

In this work, two toxin/antitoxin systems were tested in kill switch constructs with metal-ion inducible promoter P*copB*. In both cases, elevated levels of RelE-like toxins caused reduced growth rather than efficient killing of cells. Bacteriostatic rather than bactericidal effect of TA systems has been reported, and has been overcome in other suicide systems (e.g. *E. coli*) by high toxin expression (Knudsen et al., 1995; Kristoffersen et al., 2000; Wright et al., 2013). In our biocontainment system, bacteriostatic effect was likely the result of insufficient toxin levels, coupled with the presence of endogenous antitoxins that could neutralize toxin activity. Our results are in agreement with those of Cheah et al. (2013), who used the metal-ion responsive promoter P*nrsB* for induction of *E. coli*-MazF toxin in *Synechocystis* to serve as a counter-selection marker for genetic modification. Despite high inducibility of metal-ion inducible promoters, growth arrest rather than killing was observed by Ni^2+^-induction of *mazF* in their experiments, similar to what we have observed with RelE-like toxins and P*copB*.

## MATERIALS AND METHODS

### Strains and constructs

Cyanobacterium *Synechocystis* sp. PCC 6803 (WT) was used in this work. Suicide switch constructs and *lacZ*-fusions were constructed in plasmid pPMQAK1 (Huang et al., 2010). Cloning was carried out in *Escherichia coli* DH5α, and sequence-verified constructs were transferred to *Synechocystis*. Table S1 describes the parts used in suicide switch constructs and *lacZ*-fusions, the templates used for amplification, and the primers with restriction sites.

To generate suicide switch constructs, a double transcription terminator (biobrick BBa_B0015) was first inserted into the *Eco*RI site of pPMQAK1, introducing a *Nhe*I site. Kill switch constructs were assembled separately and then inserted between the *Pst*I and the new *Nhe*I site. To assemble the kill switch constructs, individual parts of the switch were first amplified by PCR (genes, promoters, RBSs, transcription terminators) or synthesized (BCD bicistronic design). Primers from Sigma-Aldrich (St. Louis, MO, USA) and Platinum *Pfx* DNA polymerase (Fisher Scientific, Pittsburgh, PA, USA) were used for PCR. Fragment synthesis was done by GeneArt Gene Synthesis (Thermo Scientific). Next, the parts were digested with restriction nucleases (Thermo Scientific), and two parts at the time were ligated with T4 DNA ligase (Thermo Scientific). The ligated parts were amplified by PCR using outer primers. Several steps of restriction, ligation and PCR were needed to assemble all parts and amplify an entire kill switch. The final kill switch composition included an additional double transcription terminator BBa_B0015 between the *Spe*I and *Pst*I sites. The last PCR product, which amplified the entire kill switch, was digested with *Pst*I and *Nhe*I and ligated into the vector. Empty vector control plasmid (EV) was obtained by deletion of the *ccdB*-containing fragment (*Xba*I-*Spe*I) from pPMQAK1.

To generate *lacZ*-fusions, the *lacZ* sequence was combined with different promoters (P*nrsB-RBS*, P*rbcL1A* and P*rnpB-RBS_1_*) by using a *Bam*HI restriction site. A double transcription terminator (biobrick BBa_B0015) was placed downstream of *lacZ* by using a *Spe*I site. The final *lacZ* composition was inserted into pPMQAK1 between *Xba*I and *Pst*I.

### Growth rate measurements and kill switch induction

Wild-type *Synechocystis* sp. PCC 6803 was grown in BG11 medium (Rippka, 1988), and strains carrying constructs in plasmid pPMQAK1 were grown in BG11 supplemented with antibiotics. Cultures were grown at 22°C under continuous light (∼28 µmol photons/m^2^/s) in flasks (gentle shaking) or in test tubes that were vortexed daily to mix bacterial suspensions. Growth was monitored by measuring optical density OD_730_ with Varian Cary 50 spectrophotometer (Agilent Technologies, Santa Clara, CA, USA).

For kill switch induction experiments, log-phase cultures (OD_730_∼0.3) were used. At least three biological replicates were performed for individual kill switch constructs. Log-phase cultures (5 ml), diluted to OD_730_∼0.03, were induced with ZnCl_2_ (12 μM final concentration), CuSO_4_ (2 μM final concentration) or were left untreated (uninduced controls), and growth was followed by measuring OD_730_. For kill switch induction in Fig. 4, BG11 plates with or without added 4 μM ZnCl_2_ were inoculated with 10-μl bacterial suspensions, and colonies were evaluated under a stereomicroscope with 25× magnification (Zeiss 476100-9901, Oberkochen, Germany).

Some kill switch inductions were carried out in Nunc 96-well plates (Thermo Scientific). Log-phase bacterial cultures (OD_730_∼0.3) growing in BG11 were used to inoculate plates. To start the growth experiment, log-phase cultures were inoculated in triplicates into wells, and inducer (for treated cells) or BG11 (for untreated cells) was added, resulting in wells containing 200 μl of bacterial suspension at cell density OD_730_∼0.03 [the inducers were prepared in BG11 medium and were added to bacteria to final concentrations of 10 or 12 μM (for ZnCl_2_) or 4 μM (for Co(NO_3_)_2_)]. Plates were incubated with gentle shaking under standard growth conditions (light, temperature), and samples were mixed daily by pipetting. Optical density (OD_600_) was followed by Sunrise™ absorbance plate reader (Tecan, Männedorf, Switzerland). On the last day of the experiment, optical density OD_730_ of KS*_PcopM195-BCD-nucA_* was measured by Varian Cary 50 spectrophotometer (Agilent Technologies), and 10-μl culture aliquots were plated on solid BG11 media.

### MTT cell viability assay

MTT analysis is used to assess cell viability by measuring reduction of yellow-colored MTT [3-(4,5-dimethylthiazol-2-yl)-2,5 diphenyl tetrazolium bromide] to purple formazan by active reductases in live cells (Li and Song, 2007). The level of reduction, which correlates with the number of living cells, can be quantified in 96-well plates by an absorbance plate reader. Kill switch cultures, wild-type cells and empty vector control cells were grown under standard growth conditions to log phase (OD_730_∼0.3). Cells were pelleted, washed 3 times with sterile dH_2_O, and final pellets were resuspended in BG11 medium. Aliquots of 100 μl (10^6^ cells) were pipetted into individual wells of a 96-well plate (BRAND*plates*^®^, BrandTech Scientific, Essex, CT, USA). To these cells, the following was added: ZnCl_2_ (to 10 or 14 μM final concentration), NiSO_4_ (to 4 μM final concentration), Co(NO_3_)_2_ (to 4 μM final concentration) or H_2_O (uninduced control samples). Treatments were carried out in triplicates. For background calculation, wells containing BG11 without bacterial cells were used. Multiwell plates containing treated cells were incubated with gentle shaking under standard growth conditions for 24 h. At that time, 20 μl of filter-sterilized MTT solution [5 mg/ml MTT in PBS (pH 6.9)] (Sigma-Aldrich) was added to each well, after which the plate was incubated in the dark at 37°C. After 3-4 h, 100 μl MTT solvent solution (1 g SDS and 8.3 μl HCl in 10 ml H_2_O) was added to each well, the plate was covered with tinfoil and incubated overnight at 28°C with gentle shaking. The next day, absorbance was read at 560 nm with a reference filter of 620 nm by Sunrise™ absorbance plate reader (Tecan). Three biological replicates were performed for each suicide switch construct. For analyzing cell viability 14 days postinduction, cells were grown and induced in test tubes (4-ml cultures) instead of multiwell plates to avoid the problem of drying. Log-phase cultures were inoculated into fresh BG11 medium in triplicates at the initial concentration OD_730_∼0.03. After being treated with ZnCl_2_ (10 μM final concentration) or H_2_O (uninduced control), cells were left to grow under standard conditions for two weeks. On day 14, 50-μl aliquots were taken from every culture (50 μl of wild-type culture contained ∼4×10^6^ cells), diluted with BG11 to 100 μl, and pipetted into 96-well plates for MTT analysis. MTT assay was then carried out as described above.

Statistical significance between the control and the kill switch cells in MTT assays was calculated using Student's two-tailed *t*-test.

### RNA isolation and RT-PCR analysis

Exponentially growing cyanobacterial cultures (60 ml, OD_730_=0.3) were used for RT-PCR analysis. Aliquots of 30 ml were induced with 10 μM ZnCl_2_ (cultures KS*_PcopM72-nucA_*, KS*_PcopM195-BCD-nucA_*, KS*_PcopB-tox0664_*) or 4 μM NiSO_4_ (culture KS*_PnrsBRBS-nucA_*), and the remaining 30 ml were left untreated. Wild-type cultures served as controls for absence of *nucA/nuiA* expression and for basal *slr0664* expression. Following a 1-h induction, the cultures were rapidly chilled, pelleted (4500 g, 10 min, 4 °C) and the cells were broken with 150-212 µm glass beads (Sigma-Aldrich). Total RNA was isolated with RNeasy Mini Kit (Qiagen, Hilden, Germany) as per manufacturer's instructions. Following removal of genomic DNA by DNase I (1 U per 1 μg RNA) (Thermo Scientific), 20 µl-reverse transcription reactions were carried out with 0.5 µg RNA (0.14 µg in case of KS*_PcopB-tox0664_*) by using RevertAid First Strand cDNA Synthesis Kit (Thermo Scientific) per manufacturer's instructions. Reactions containing cDNA were diluted 1:1 with dH_2_O and used for PCR with DreamTaq DNA polymerase (Thermo Scientific) following manufacturer's protocol (1.5 µl cDNA was used as template for KS*_PcopM72-nucA_* and KS*_PcopM195-BCD-nucA_*; 2.5 µl for KS*_PcopB-tox0664_*; and 3 µl for KS*_PnrsBRBS-nucA_*). Control reverse transcription reactions without added reverse transcriptase were used for PCR-verification of successful (prior) removal of genomic DNA, and PCR reactions without template were used to verify absence of reagent contamination. PCR primers were: for *nucA* (5′-CCATCAATCAGCGTGCATTTAC-3′ and 5′-TGTTGTTGTAGGAGAGTGCATATT-3′); for *nuiA* (5′-GAGTGAGTCTGAATACCCATTTGA-3′ and 5′-TCTTGTCCGTGACCTGTTTG-3′); for *slr0664* toxin (5′-GCAAAAAGGCGTCATTAGT-3′ and 5′-AATCGGTAGCCAGAACTTT-3′); for control housekeeping gene *rnpB* (Pinto et al., 2012) (5′-CGTTAGGATAGTGCCACAG-3′ and 5′-CGCTCTTACCGCACCTTTG-3′).

### Beta-galactosidase assay

To measure promoter activity in *Synechocystis*, we made modifications to a previously described beta-galactosidase protocol (Miller, 1972). Log-phase cultures harboring reporter constructs (10 ml) were treated with NiSO_4_ (4, 10, 16 or 27 μM final concentration), Co(NO_3_)_2_ (8 μM final concentration), ZnCl_2_ (8 μM final concentration) or H_2_O (uninduced control samples) for 18 h and harvested by centrifugation (12,500 ***g***, 4°C, 10 min). Pellets were resuspended in 1 ml of ice cold buffer Z (60 mM Na_2_HPO_4_, 40 mM NaH_2_PO_4_, 10 mM KCl, 1 mM MgSO_4_, pH 7), which was freshly supplemented with 2-mercaptoethanol (50 mM final concentration) and EDTA-free Pierce protease inhibitors (1× final concentration) (Fisher Scientific). Chloroform (100 μl) was added to the samples (use of SDS was avoided due to interference with subsequent Bradford assay). About 300 μl of 150-212 µm glass beads (Sigma-Aldrich) were added and cells were broken by vortexing (10 times for 30 s, with 1-min incubations on ice in-between). After centrifugation (10 min, 4°C, maximal speed), about 1 ml of aqueous phase was collected. Two 200-μl aliquots were used to determine total protein concentration by Bradford assay and the rest of the aqueous phase was used to measure beta-galactosidase activity. Triplicate cell aliquots (from 2-200 μl, depending on expected strength of promoter response) were diluted with buffer Z to 400 μl and incubated at 28°C for 2 min. Initial reaction time was recorded when 72 μl of ONPG [o-nitrophenyl-β-D-galactoside; 4 mg/ml solution in phosphate buffer (60 mM Na_2_HPO_4_, 40 mM NaH_2_PO_4_, pH 7)] was added to each of the three samples. Reaction in one sample (blank sample) was immediately terminated by addition of 176 μl of 1 M Na_2_CO_3_. The two other samples were terminated simultaneously when they developed sufficient yellow color, and termination time was recorded. After centrifugation (5 min, maximal speed), samples (500 μl) were diluted with Milli-Q water (500 μl), transferred to UV-Vis cuvette, and absorbance at 420 and 550 nm (A_420_ and A_550_) was measured against the blank sample by Varian Cary 50 spectrophotometer (Agilent Technologies). Promoter activity was calculated from the modified equation, which gives standardized Miller Units, (s)MU:
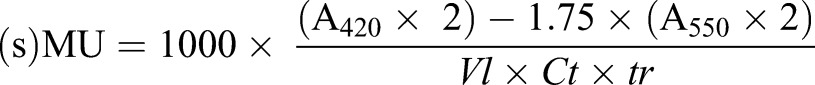
where *Vl* is the volume of cell lysate used (ml), *Ct* is the total protein concentration (μg/ml) and *tr* is the reaction time (min). Absolute unit of (s)MU is given as μmol of ONP (o-nitrophenol) product per min per μg of total protein. Three biological replicates were performed for each reporter construct.
